# Juvenile Recurrent Parotitis: The Role of Sialendoscopy

**DOI:** 10.1155/2019/7278907

**Published:** 2019-09-29

**Authors:** Efimia Papadopoulou-Alataki, Panagiotis Dogantzis, Angelos Chatziavramidis, Sofia Alataki, Panagiota Karananou, Kyriaki Chiona, Iordanis Konstantinidis

**Affiliations:** ^1^4th Department of Pediatrics, Aristotle University of Thessaloniki, School of Medicine, Papageorgiou General Hospital, Ring Road Nea Efkarpia 56403, Thessaloniki, Greece; ^2^Sialendoscopy Clinic, 2nd Department of Otolaryngology, Aristotle University of Thessaloniki, School of Medicine, Papageorgiou General Hospital, Ring Road Nea Efkarpia 56403, Thessaloniki, Greece

## Abstract

Juvenile recurrent parotitis (JRP) is a recurrent parotid inflammation of nonobstructive, nonsuppurative nature. It manifests in childhood and usually resolves after puberty but may also persist into adulthood. JRP is characterized by recurrent episodes of unilateral or/and bilateral parotid swelling with pain, reduction of salivary secretion, swallowing difficulty, fever, and malaise. The cause of this condition remains obscure. Throughout the last two decades, many therapeutic methods have been used in order to reduce the frequency and severity of JRP. During the acute episodes, conservative approaches (antibiotics, analgesics, sialogogues, massage of the parotid gland, and mouth rinses) are used. Parotidectomy has been suggested in rare selective occasions. Recently, a promising concept of sialendoscopy, which is a minimal invasive endoscopic technique, has been applied. This review outlines the literature on JRP focusing on methods and challenges in diagnosing JRP along with the differential diagnosis of JRP and the function of the parotid during JRP. In addition, we describe the treatment options for JRP, pointing out the importance of sialendoscopy as a diagnostic and treatment procedure that offers improvement in patients' daily life.

## 1. Introduction

The term juvenile recurrent parotitis (JRP) is used to describe a parotid inflammation of children and adolescents which is nonobstructive and nonsuppurative in nature. It is a rare salivary gland disease presenting as parotid swelling that appears at least twice before adolescence. It is usually self-limiting during the second decade of life. After mumps, JRP is the most common inflammatory disease of the salivary glands in childhood, especially during the times before the universal vaccination against mumps. [1].

Saliva plays an important role in swallowing, digestion, speech, taste, and oral health. JRP when persistent could lead to chronic salivary dysfunction with severe sequelae. Conservative therapeutic approaches during the acute episodes (antibiotics, analgesics, sialogogues, massage of the parotid gland, and mouth rinses) are still used. Surgical interventions, e.g., parotidectomy and duct ligation, have also been suggested [2]. Nowadays, minimally invasive interventions both as diagnostic and therapeutic tools are applied.

The aim of this review is to present current assessment and treatment strategies and a view of current literature relevant to JRP in children and adolescents.

## 2. Clinical Presentation

Juvenile recurrent parotitis is presented with recurrent episodes of swelling of the parotid salivary gland. It is characterized by acute unilateral or bilateral parotid inflammation. Pain, erythema, local rise of temperature, fever, and malaise can be present as well [1]. Salivary secretion is reduced leading to swallowing difficulty. At examination, nonpalpable jaw angle and discharge of mucopurulent saliva through the duct papilla upon compression of the gland are frequently observed.

The symptoms usually last 2–7 days with a median of 3 days or can persist for weeks [3, 4]. The age of onset appears to have two peaks, one at 2–5 years and one at the age of ten [3]. It is found to be more common in male rather than female [3]. The symptoms' recurrence varies depending on study as much as 30 times annually, but the mean number of episodes is about 1.5 per year [4].

## 3. Etiopathology

The etiopathology of JRP is still unidentified. Many theories as to what may be the underlying cause have been suggested throughout the last twenty years. Furthermore, quite a few factors are considered responsible for the susceptibility of the parotid to develop JRP. A congenital malformation of Stensen's duct can increase the possibility of a parotid infection originating in the oral cavity. Dental malocclusion and bad oral hygiene can serve as a predisposing factor. Immunological deficits such as IgA deficiency and/or IgG subclass deficiency and HIV infection have also been implicated. Allergy, systematic, and autoimmune diseases like sarcoidosis and Sjögren's syndrome have been considered too [1, 3]. Moreover, the theory of JRP being an immunopathological disorder of the mucosa-associated lymphoid tissue (MALT) and a predecessor of a benign lymphoepithelial lesion has been suggested. This theory is supported by histological studies of different stages of chronic recurrent parotitis, which show progressively increasing periductal inflammation and formation of lymph follicles alongside the destruction of the normal structure of the parotid [5].

### 3.1. Function of the Parotid during Juvenile Recurrent Parotitis

Saliva is of paramount importance to every function the mouth and oral cavity are responsible for. It contains enzymes such as amylase and lipase that are important for digestion of food. It provides taste and lubrication of the oral cavity. It helps create and swallow the food bolus and also partakes in the proper articulation of words modulating speech. Saliva's antimicrobial properties could not be overlooked. With the use of secretory IgA immunoglobulin, mucins, lactoferrin, and lactoperoxidase that it contains, saliva keeps the balance between the normal flora of the oral cavity and microbial colonization. In addition, saliva retains steady the natural pH of the oral cavity [6].

In JRP, due to the inflammation of the parotid, the function of the gland is affected based on quantity and quality of saliva changes. During JRP, the inflammatory cycle diminishes salivary flow leading to inflammation, which in turn promotes ductal metaplasia, followed by amplified mucinous secretion and then decreased salivary flow, reinforcing a vicious cycle [7]. Predisposing factors of salivary gland inflammatory cycle are dehydration that diminishes saliva flow, infection that induces inflammation, congenital ductal abnormalities, and autoimmune duct destruction affecting ductal histology [7]. Xie at al. found that serum amylase activity can be used to assess the function of the salivary glands and furthermore can be used as a diagnostic marker [8]. If JRP is left untreated allowing the inflammation of the parotids to get extensive, xerostomia (dry mouth) could be the aftermath [9]. Usually the symptoms of xerostomia become noticeable only after the salivary output is reduced by 50%. This apart from the feeling of dry mouth can mean cracked, atrophic, and dehydrated lips. The tongue can appear reddened with absence of papillation. Dental decay and caries as well as candidiasis and other oral infections can also occur. The reduction of saliva could also lead to eating disorders and malnutrition as well as speaking difficulties and malarticulation. The dry oral mucosa is also susceptible to pain when it comes in contact with spicy or coarse foods [6]. A patient with JRP is suffering from the symptoms of the acute phase like pain, fever, and malaise, but is also in danger of all the manifestations of xerostomia. These along with painful swelling cause disability and hinder socialization, attendance to school and nonschool associated activities, especially in childhood.

## 4. Diagnosis

Recently, diagnostic criteria for JRP are suggested. In the meta-analysis of Garavello et al. [10] inclusion criteria: age <16 years, recurrent unilateral or bilateral swelling and at least 2 episodes during the last 6 months as well as exclusion criteria: obstructive lesions, dental malocclusion, Sjögren syndrome, and IgA deficiency are proposed [10].

In general, the diagnosis of juvenile recurrent parotitis is mostly clinical. It is based on personal history, physical examination, and laboratory investigation and is confirmed with imaging methods.

### 4.1. Imaging

#### 4.1.1. Sialography

Sialography has been the primary imaging method to diagnose JRP. It is used to depict the anatomical structure of the parotid duct and detect any anatomical malformation by inserting sialographic dye in the ducts. This procedure is invasive, requires expertise from the operator and the cooperation of the patient which sometimes is impossible. The use of iodizing radiation should also be considered as a drawback since this technique is performed on children and adolescents [9].

#### 4.1.2. Ultrasonography

**Figure 1:**
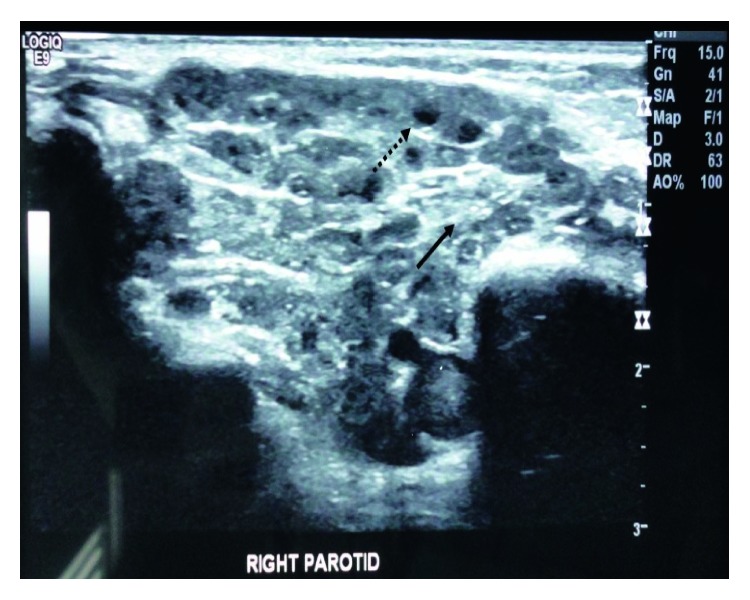
Ultrasonography image showing the parotid gland with low, heterogeneous echogenicity (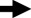), and multiple hypoechogenic areas (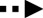).

The use of ultrasonography in order to diagnose JRP is a topic well considered and applied in everyday clinical routine. The parotid presents with an enlarged heterogeneous ultrasound picture, either uni- or bilaterally. Areas of hypoechogenicity of 2–4 mm can be observed which could correspond to either sialectasia or lymphocytic infiltration (Figure 1). Enlarged intraglandular lymph nodes and microcalcifications are also found [11]. The consistency of the gland remains abnormal even during the dormant periods. This method is also useful and efficient for the follow-up of the patients. Quite accessible, noninvasive, easily repeatable, cheap, and fast are the advantages that have made ultrasonography the first-line diagnostic imaging tool for JRP although it relies on the expertise of the operator [12, 13].

#### 4.1.3. Magnetic Resonance Imaging and Magnetic Resonance Sialography

Magnetic resonance imaging (MRI) and magnetic resonance (MR) sialography have also been used as imaging techniques of the parotid in JRP. Major advantage of the combination of these techniques is the evaluation of both the ductal (via MR sialography) and the parenchymal (via MRI) parts of the parotid. With the aforementioned noninvasive techniques, the difference between chronic and acute inflammation can be seen by comparing the intensity of the signal of the pathologic gland with that of the opposite side. Furthermore, MR sialography can discern sialectasia from focal lesions which can be used to differentiate JRP with infiltrative diseases like lymphoma. Although these methods are noninvasive and with no radiation, it must be pointed out that they are expensive and not for everyday practice as the patient needs to be sedated in order to be carried out [14].

#### 4.1.4. Computed Tomography

Computed tomography can assess the parotid parenchyma as well as neighboring structures. Glandular enlargement and enhancement which can be correlated with parotitis and other focal lesions as sialoliths can be depicted with the implementation of computed tomography. Nevertheless, the use of iodizing radiation should be considered [9].

#### 4.1.5. Sialendoscopy

**Figure 2:**
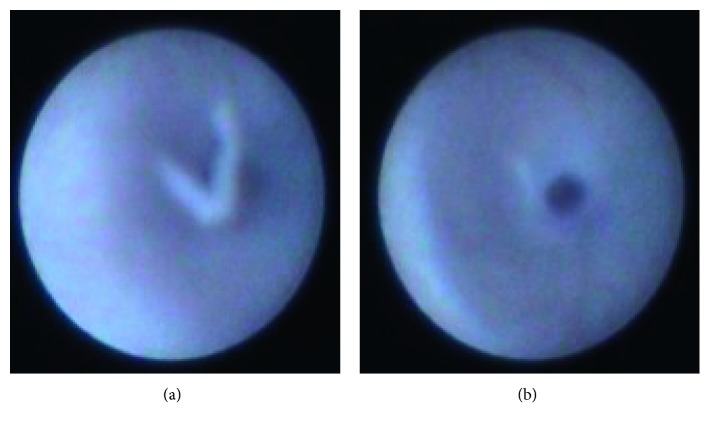
Endoscopic view of the parotic duct with the typical white appearance. (a) Debris partially obstructing the lumen. (b) Ductal stenosis.

Sialendoscopy is a newer technique implemented in the diagnosis of salivary diseases. It is a minimally invasive method using a semirigid optic endoscopic device. By inserting it through the oral cavity and papilla catheterization, it is possible to observe the anatomical course of Stensen's duct and any pathology involved with it. Sialoliths, sialectasia, and stenosis of the duct can be detected [15]. The most diagnostic sialendoscopic findings are the white ductal wall appearance and scarce vascularity in the ductal lumina along with confined/diffused stenosis and many fibrinous debris/mucous plugs that are seen very often in children (Figure 2) [1, 16].

Advantages of this diagnostic method are its noniodizing nature, low morbidity, and the fact that it can not only be used to diagnose JRP, but it can be used to treat it at the same time [17].

The only limitation of the procedure is the need of anesthesia. The option of general or local anesthesia depends on age. Almost all the doctors perform the procedure under general anesthesia [4, 18–22].

In the recent literature, only a few studies refer to the use of local anesthesia in sialendoscopy. Konstantinidis et al. [15] and Papadopoulou-Alataki et al. [1] reported favorable outcome of sialendoscopy under local anesthesia in children and adolescents >8 years of age [1, 15]. Jokela et al. [23] published very encouraging results on the tolerability of sialendoscopy under local anesthesia in adult patients with JRP [23].

## 5. Differential Diagnosis

A variety of pediatric and other diseases should be taken into account. We should firstly consider that an infection could be the underlying cause of the symptoms that were described. Alongside mumps, that became rare through the universal vaccination in infancy, other viruses and also bacteria can cause infectious parotitis. Apart from infection, autoimmune diseases can be associated with parotid swelling as much as neoplasms, anatomical differences, and others.  Table 1 summarizes the causes that should be excluded before the diagnosis of JRP [6].

The assistance provided to the diagnosis by laboratory exams is of paramount importance. The need to differentiate all the possible causes of parotid swelling calls for the proper laboratory investigation in order to have no doubt about the consistency of the diagnosis. The standard blood and biochemical tests alongside with erythrocyte sedimentation rate (ESR) and C-reactive protein (CRP) should be implemented. Special immunologic tests containing C3 and C4 serum levels and immunoglobulin levels should also be performed. Serological testing for mumps and for the rest of the possible infectious causes of parotid swelling is advised. Sample from Stensen's duct papilla should also be taken to facilitate cultures in order to identify a potential microbial infection. Immunological testing should also include autoantibodies, antinuclear antibodies (ANA), and anti-double stranded deoxyribonucleic acid antibodies (anti-dsDNA) for systematic lupus erethematicus (SLE), SS-A/anti-Ro, SS-B/anti-La antibodies for Sjögren's syndrome.

Imaging can clarify parenchymal anomalies like neoplasms, genetic defects, or pathology of the surrounding structures like the rare case of mandibular osteomyelitis that can mimic JRP [24–26]. Salivary stones lodged into the salivary duct (sialolithiasis) should be included in the differential diagnosis as well. Imaging and sialendoscopy can help clarify the cause in those instances [27].

It is very important that every patient with recurrent parotid swelling in childhood or adolescence to be screened and managed by a pediatrician first before referring to any endoscopic intervention [28].

## 6. Treatment Strategies

Throughout the years, many treatment options were proposed and exploited either invasive or noninvasive. Initial treatment of the acute episode starts with symptomatic treatment: antibiotics, warm compresses and massaging of the parotid, sialagogues, and hydration. Analgesics and anti-inflammatory drugs such as nonsteroidal anti-inflammatory drugs are used to treat the pain and reduce the inflammatory process. Another evidently different therapeutic method that was proven to work preventing the recurrences of JRP is that of the traditional Chinese medicine using bear bile and Huangqi [29]. However, the clinical application of this method needs to be confirmed by an independent trial, before it starts being used widely.

To reduce the likelihood of recurrent episodes arising and to treat those cases that failed to subside with the original treatment, some other treatment methods were included, such as intraductal injection of a sclerosing substance or corticosteroids, the dissection of the tympanic nerve, parotid duct ligation, and radiation. A treatment method that was once considered the golden standard for the treatment of severe JRP was parotidectomy. Although it was effective in treating JRP, there were many risks and potential side effects associated with this method such as the injury of the facial nerve, bad aesthetic outcome, earlobe numbness, and Frey's syndrome [4, 30]. That is why this technique along with radiation and neurectomy is not used in routine for the treatment of JRP any more except in strictly selected cases [30].

### 6.1. Sialendoscopy

During the last two decades a promising endoscopic technique was established for JRP treatment. It was described by Nahlieli et al. in 1997, initially for the treatment of sialolithiasis [27]. Sialendoscopy is a diagnostic and simultaneous treatment modality. This new procedure provides irrigation and dilatation of Stensen's duct under direct vision [18].

Sialendoscopy as treatment procedure in JRP was widely accepted because it is a safe and minimally invasive procedure with the purpose of diminishing the rate of recurrences for parotid inflammation in children [31]. It is becoming the first-line treatment for children with JRP [7]. The procedure is used to visualize the canal system of Stensen's duct and examine it for impeding agents such as sialoliths or mucosal debris, tight areas of the duct (stenosis), and whitish ductal mucosa (evidence of inflammation) [11, 31]. The procedure is usually performed with the patient in supine position with the head slightly elevated [32]. After the identification of the papilla, the endoscope is gently inserted through the papilla and pressed forward slowly and carefully into the ductal system while steady irrigation is used to dilate the ductal system. Once inside, through the endoscope work canal, steroids, antibiotics, or a combination of the two is injected to the dilated salivary duct. The ductal system can also be cleansed by rinsing with saline solution and washing out any debris or small stones that might cause the swelling. However, it is not clear just yet whether the dilation of the duct, the drugs injected into it, or lavaging of the duct is definitively efficacious [32].

Although most cases are performed under general anesthesia, recent inclination tends to sialendoscopy under local anesthesia, where a 4% xylocaine drenched swab is placed on the papilla of the duct for about ten minutes before the procedure [15]. There is no consensus whether local or general anesthesia should be standardized depending on every centre's experience. Under general anesthesia, the benefits are certain analgesia during the operation, keeping a safe and open airway at all times, and no need for patient's cooperation. On the other hand, local anesthesia is less invasive making the recovery time and the duration of the operation shorter and rendering the procedure cheap and simple. However, intricate procedures with challenging anatomical conditions and patients who refuse to cooperate should be considered for general anesthesia. Konstantinidis et al. [15] and Papadopoulou-Alataki et al. [1] conclude that sialendoscopy under local anesthesia could be offered to subjects >8 years in outpatient setting, indicating significant improvement in patients' activity and social life [1, 15].

Complications regarding this method include ductal perforation, intraoperation complications, postoperative ductal stenosis, and airway obstructions amongst others. As for the effectiveness of sialendoscopy, the full potential of the method has yet to be reached. Physicians are yet not fully comfortable with the use of such small endoscopes and fear the possible complications [32]. Garavello et al. [10], in a systematic review on JRP treatment studies state that the rate of recurrences after sialendoscopy was 25.8%, and their severity was lower [10]. However, JRP might have resolved itself in some of the cases, as the rate of spontaneous resolution is not yet known, indicating that the most suitable management is still unknown [10].

## 7. Prognosis

Juvenile recurrent parotitis is mostly a self-limiting condition that affects children until late adolescence with very few cases continuing in adulthood as chronic parotitis. As mentioned before, after sialendoscopy most cases were resolved. Those few cases that were unresponsive to treatment would eventually lead to complete lymphatic transformation of the gland that may require total parotidectomy as last resort [33]. Therefore, the need to identify the most suitable treatment for JRP remains crucial.

## 8. Conclusion

Juvenile recurrent parotitis is a condition that has still some issues to be solved. The need for more studies to find out the rate of the diseases' spontaneous resolution seems to be clear. More randomized trials are needed to determine the best method to treat JRP and to clarify the factors indicating which method will be applied for individualized cases. For now, sialendoscopy seems to be the key in understanding, diagnosing, and treating JRP and is considered to be the modern challenging solution to relieve the recurrences of JRP.

## Figures and Tables

**Table 1:** Differential diagnosis of juvenile recurrent parotitis.

*Infectious diseases*	
Bacterial	Staphylococcus aureus, group B streptococcus, Mycobacterium tuberculosis, Mycobacterium avium
Viral	Mumps, adenovirus, HIV, EBV, CMV, parvo B19, influenza/parainfluenza virus, coxsackievirus

*Autoimmune diseases*	Sjögren's syndrome, systemic erythematosus lupus

*Neoplasms*	
Benign	Pleomorphic adenoma, papillary cystadenoma, basal cell carcinoma, canalicular adenoma, oncocytoma
Malignant	Polymorphic low-grade adenocarcinoma, mucoepidermoid carcinoma, cystadenocarcinoma, adenoid cystic carcinoma, salivary duct carcinοma, epithelial-myoepithelial carcinoma, squamous cell carcinoma of salivary origin, lymphoma

*Others*	Sarcoidosis, cystic fibrosis, selective IgA deficiency, sialolithiasis, toxoplasmosis, sialocele, mikulicz syndrome, metabolic disorders, mandibular osteomyelitis

HIV: human immunodeficiency virus; EBV: Epstein–Barr virus; CMV: cytomegalovirus.
